# Antifibrogenic Influence of *Mentha piperita* L. Essential Oil against CCl_4_-Induced Liver Fibrosis in Rats

**DOI:** 10.1155/2018/4039753

**Published:** 2018-04-19

**Authors:** Hanan A. Ogaly, Nadia A. Eltablawy, Reham M. Abd-Elsalam

**Affiliations:** ^1^Department of Chemistry, College of Sciences, King Khalid University, Abha, Saudi Arabia; ^2^Biochemistry Division, National Organization for Drug Control and Research (NODCAR), Giza, Egypt; ^3^Department of Pathology, Faculty of Veterinary Medicine, Cairo University, Giza, Egypt

## Abstract

Essential oils of some aromatic plants provide an effective nonmedicinal option to control liver fibrosis. *Mentha piperita* L. essential oil (MPEO) have been reported to possess protective effects against hepatotoxicity. However, its effect against liver fibrosis remains unknown. The present study investigated the antifibrogenic potential of MPEO and its underlying mechanisms. Forty male rats divided into 4 groups were used: group 1 served as normal control, group 2 (liver fibrosis) received CCl_4_ (2.5 mL/kg, IP, twice weekly) for 8 weeks, group 3 concurrently received CCl_4_ plus MPEO (50 mg/kg, IP, daily, from the 3rd week), and group 4 received MPEO only. MPOE significantly improved the liver injury markers, lipid peroxidation (LPO), antioxidant capacity, CYP2E1 gene expressionand liver histology. Furthermore, MPOE ameliorated liver fibrosis as evidenced by the reduced expression of desmin, *α*-smooth muscle actin (*α*-SMA), transforming growth factor-*β*1 (TGF-*β*1), and SMAD3 proteins. In addition, MPOE counteracted the p53 upregulation induced by CCl_4_ at both mRNA and protein levels. In conclusion, MPOE could effectively attenuate hepatic fibrosis mainly through improving the redox status, suppressing p53 and subsequently modulating TGF-*β*1 and SMAD3 protein expression. These data promote the use of MPOE as a promising approach in antifibrotic therapy.

## 1. Introduction

Liver fibrosis and cirrhosis are the ultimate consequences of chronic hepatic injury induced by various etiological agents [1]. The mechanism of liver fibrosis has been extensively studied. However, effective antifibrotic therapies are lacking [2]. The pathogenesis of hepatic fibrosis is generally based on the activation of HSCs and excessive extracellular matrix (ECM) production [2]. Oxidative stress plays a central role in triggering these inflammatory and fibrotic responses [3]. Among the various signaling pathways involved in pathogenesis of liver fibrosis, TGF-*β*1/SMAD is considered the most significant signaling pathway [4]. Therefore, inhibiting TGF-*β*1 was found to be efficient in attenuating liver fibrosis [5, 6]. The tumor suppressor p53 is another important cell signal primarily stimulated in response to oxidative damage and oncogene activation [7]. Accumulating evidences suggested the involvement of p53 in the pathophysiology of various nontumoral fibrotic liver diseases in both human and animals [8, 9]. These data suggest that p53 regulation could serve as an important therapeutic target for fibrotic liver diseases.

Medicinal plants and their derivatives contain a wide variety of bioactive phytochemicals with a diverse pharmacological spectrum [10, 11]. Essential oils constituents including terpenes, terpenoids, phenylpropenes, and other degradation products have been reported to exhibit strong antioxidant and anti-inflammatory activities [12]. The effectiveness of some essential oils to alleviate the hepatotoxicity [13] and hepatic fibrosis [14] has been proven. *Mentha piperita* L. (peppermint) is one of the most popular and widely used herbs. Pharmacological investigations have demonstrated that *M. piperita* possesses analgesic, antifungal [15], antibacterial [16], antiparasitic, and immunomodulatory activities [17]. Moreover, the hepatoprotective effects of *M. piperita* leaves extract [18], oil [19, 20], or its active components menthol and menthone [21] have been reported. However, to the best of our knowledge, the effect against hepatic fibrosis has not been reported. Therefore, the present study aimed to investigate the effects of *Mentha piperita* L. essential oil (MPEO) against hepatic fibrosis and to elucidate the potential underlying molecular mechanisms.

## 2. Material and Methods

### 2.1. MPOE Preparation and Characterization

*Mentha piperita* L. leaves were purchased from Harraz Drug stores (Cairo, Egypt). A voucher specimen of the studied plant material was deposited at Biochemistry Department, National Organization for Drug Control and Research (NODCAR), Egypt. Essential oil was extracted and characterized for its chemical composition using gas-liquid chromatography coupled to mass spectrometry (GC-MS) as described by Ogaly et al. [14].

### 2.2. Experimental Design

Adult male rats (150–170 g) were obtained from the breeding unit of the Research Institute of Ophthalmology (Giza, Egypt). Rats were housed under constant temperature and 12 h light/dark cycle with free access to water and standard chow diet. All animal procedures were performed according to the protocol approved by the Institutional Animal Care and Use Committee (IACUC), Cairo University (CU-II-F-1-18).

Forty rats were randomly divided into four groups. Group 1 (control) was given corn oil (2 mL/kg, IP). Group 2 (fibrosis model) was given CCl_4_ 1 : 4 mixture with corn oil (2.5 mL/kg, IP) twice weekly for eight weeks. Rats in group 3 received CCl_4_ for two weeks to establish liver injury and fibrosis and then treated with MPOE (50 mg/kg, IP), daily from the 3rd to 8th week. Group 4 received MPOE (50 mg/kg, IP) from the 3rd to 8th week. The selected dose for MPOE (50 mg/kg) was previously reported to be hepatoprotective for rats [20].

### 2.3. Samples Collection

At the end of experiment, animals were anesthetized with ethyl ether. Blood samples (3-4 mL) were collected by retro-orbital puncture, and serum was separated by centrifugation (4000 rpm/10 min). Afterward, all animals were sacrificed by cervical dislocation under ethyl ether anesthesia for humane reasons; the whole liver was immediately removed, rinsed in cold normal saline, and kept at −20°C until further analyses. Liver homogenates (10%) were prepared in 0.1 M ice-cold phosphate buffered saline (pH 7.4) followed by centrifugation at 14,000 ×g, 15 min at 4°C. The separated supernatants were kept at −20°C. For histopathological examination, parts of liver were fixed in 10% neutral buffered formalin and underwent routine processing for paraffin embedding.

### 2.4. Biochemical Analyses

Serum samples were used to measure liver injury markers alanine aminotransferase (ALT) and aspartate aminotransferase (AST) according to the method of Reitman and Frankel [22]. Liver homogenates were used for measurement of nitric oxide (NO) [23], malondialdehyde (MDA) as a thiobarbituric acid reactive substance (TBARS) [24], superoxide dismutase (SOD) activity [25], catalase (CAT) activity [26], reduced glutathione (GSH) level [27], and total antioxidant capacity (TAC) using commercial kits (Biodiagnostic, Cairo, Egypt). Total protein content was measured according to the method of Bradford [28].

### 2.5. Histopathological Analyses

Seven liver samples were harvested from each experimental group and fixed in 10% neutral buffered formalin and then processed to obtain 4 *μ*m paraffin-embedded sections. The sections were stained with hematoxylin and eosin (H&E) and Masson's trichrome (MT). MT staining was performed to assess collagen fibers distribution and to determine liver fibrosis %. A numerical scoring system [29] was performed to assess the grade of fibrosis, as follows: 0, no fibrosis (normal); 1, fibrous expansion of some portal areas; 2, fibrous expansion of most portal areas; 3, fibrous expansion of most portal areas with occasional portal-to-portal bridging; 4, fibrous expansion of portal areas with marked bridging (portal to portal as well as portal to central); 5, marked bridging (portal to central as well as central to central) with occasional nodules formation; and 6, cirrhosis.

### 2.6. Immunohistochemical Analyses

The immunohistochemical (IHC) analyses were done according to the methods of Ogaly et al. [14]. Briefly, tissue sections were deparaffinized and rehydrated. The antigen retrieval was performed by pretreating the tissue sections for 20 min with citrate buffer pH 6 at microwave oven. Sections were incubated with rabbit polyclonal antibody to *α*-SMA diluted 1 : 200 (ab5694; Abcam, Cambridge, UK), rabbit polyclonal antidesmin antibody diluted 1 : 200 (ab15200; Abcam, Cambridge, UK), rabbit polyclonal anti-TGF-*β*1 antibody with concentration of 20 *μ*g/mL (ab92486; Abcam, Cambridge, UK), rabbit polyclonal anti-SMAD3 antibody diluted 1 : 100 (ab28379; Abcam, Cambridge, UK), and rabbit polyclonal anti-Tp53 antibody diluted 1 : 100 (ab131442; Abcam, Cambridge, UK) for two hours in a humidified chamber. The tissue sections were incubated with goat antirabbit IgG H&L (HRP) (ab205718; Abcam, Cambridge, UK). Finally, the slides were incubated for 10 min with 3,3′-diaminobenzidine tetrahydrochloride (DAB; Sigma) as chromagen, counterstained with hematoxylin, and mounted with DPX. The image analyses of the stained sections were performed by Leica Qwin 500 Image Analyzer (Leica, Cambridge, England). In each group, seven sections were examined. The percentage of the immunopositive area (%) was calculated as mean of 10 fields/section.

### 2.7. Gene Expression Analyses

Total RNA isolated from liver tissues using RNeasy Mini Kit (Qiagen) was reverse transcribed and subjected to quantitative real-time RT-PCR as previously described [14]. mRNA expression levels of Tp53 and CYP2E1 genes were assessed using GAPDH gene as the reference gene. Briefly, cDNA was added to a Quantifast SYBR Green qPCR Master Mix (Qiagen) containing 30 pg/mL of each primer (Table 1). The thermal program included 40 cycles of denaturation at 95°C for 15 s, annealing at 60°C for 15 s, and extension at 72°C for 45 s. The first denaturation was extended to 1 min. Calculation of gene expression was done following Livak and Schmittgen [30].

### 2.8. Statistical Analysis

SPSS version 16.0 statistical package was used to analyze the data. All data were expressed as mean ± standard error (SE). One-way analysis of variance (ANOVA) was used to assess the differences between groups. Difference was considered statistically significant at *p* < 0.05 by Duncan's multiple comparisons.

## 3. Results

### 3.1. Chemical Composition of MPEO

**Figure 1:**
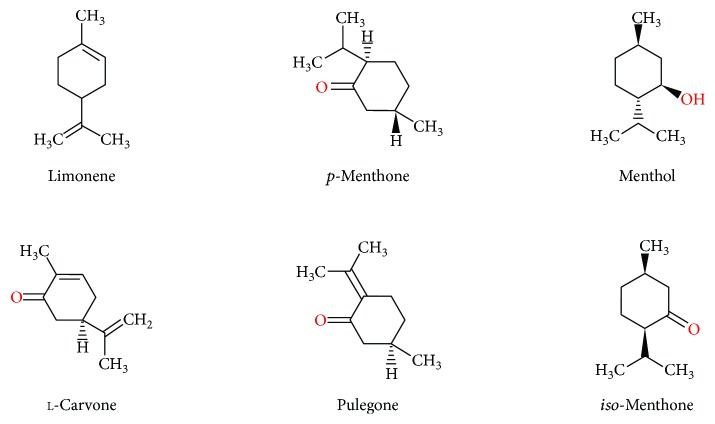
Antioxidant volatile constituents found in *Mentha piperita* essential oil.

Hydrodistilled MPEO was subjected to qualitative and quantitative analyses using gas chromatography coupled with mass spectrophotometry (GC-MS). Ten chemical constituents could be identified by elution on HP-5872 column (Table 2). The major constituents of MPEO, in order of their percentage (Figure 1), were menthol (46.7%), *p*-menthone (18.3%), l-carvone (15.2%), pulegone (6.3%), *iso*-menthone (5.3%), and d-camphor (3.3%). Small amounts of 1,8-cineol, beta pinene, *trans*-caryophyllene, and limonene were also identified.

### 3.2. MPEO Improves Liver Functions in CCl_4_-Induced Liver Fibrosis

As shown in Table 3, CCl_4_-intoxicated rats (group 2) showed a severe increase in liver marker enzymes, ALT and AST, to about 62- and 23-folds, respectively, compared to the control group. MPOE coadministration (group 3) exerted a significant reduction of ALT and AST levels to 76.8% and 60.4%, respectively, as compared to the CCl_4_ group. There were no significant differences in liver enzymes between groups 1 and 4 (Table 3).

### 3.3. MPEO Attenuates Oxidative Stress in CCl_4_-Induced Liver Fibrosis

CCl_4_ caused a marked disruption of oxidant/antioxidant balance in liver as indicated by the statistically significant elevation in MDA and NO levels to 236% and 407% in group 2 as compared to the control group (Table 3). Besides, there was a marked reduction in SOD and CAT antioxidant enzymes activities with a dramatic depletion in hepatic GSH content to 24.4%, 43.2%, and 23.4% of the control levels, respectively (Table 4). In the same line, TAC of the liver of CCl_4_-intoxicated group showed a significant reduction to 39.7% (Table 4). Administration of MPOE concurrently with CCl_4_ for 6 weeks (group 3) showed a significant reduction of MDA and NO levels compared to those of group 2 (Table 3). MPOE partially restored SOD and CAT activities and GSH level (Table 4). Improvement of CCl_4_-induced oxidative stress by MPOE was confirmed by the significant elevation of TAC, reaching 74% of the normal control level (Table 4). There were no significant differences in all measured oxidative stress markers or antioxidant parameters between groups 1 and 4 (Tables 3 and 4).

### 3.4. MPEO Ameliorates Fibrotic Alterations in CCl_4_-Induced Liver Fibrosis

**Figure 2:**
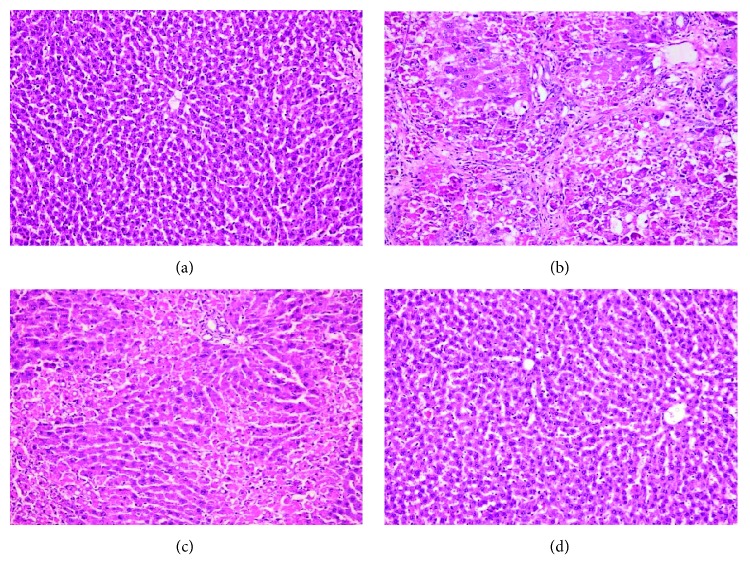
Histopathological examination of the liver tissues (H&E, ×200). (a) Group 1 (normal control) showing normal histological picture of the liver. (b) Group 2 (fibrosis control) showing fatty degeneration of the hepatocytes, hepatocellular necrosis, and mononuclear inflammatory cells aggregation along the collagenous septa. (c) Group 3 (MPEO-treated) showing moderate hepatocellular necrosis, marked reduction of collagenous septa formation and mononuclear inflammatory cells aggregation. (d) Group 4 (MPEO control) showing normal hepatic cellular architecture.

**Figure 3:**
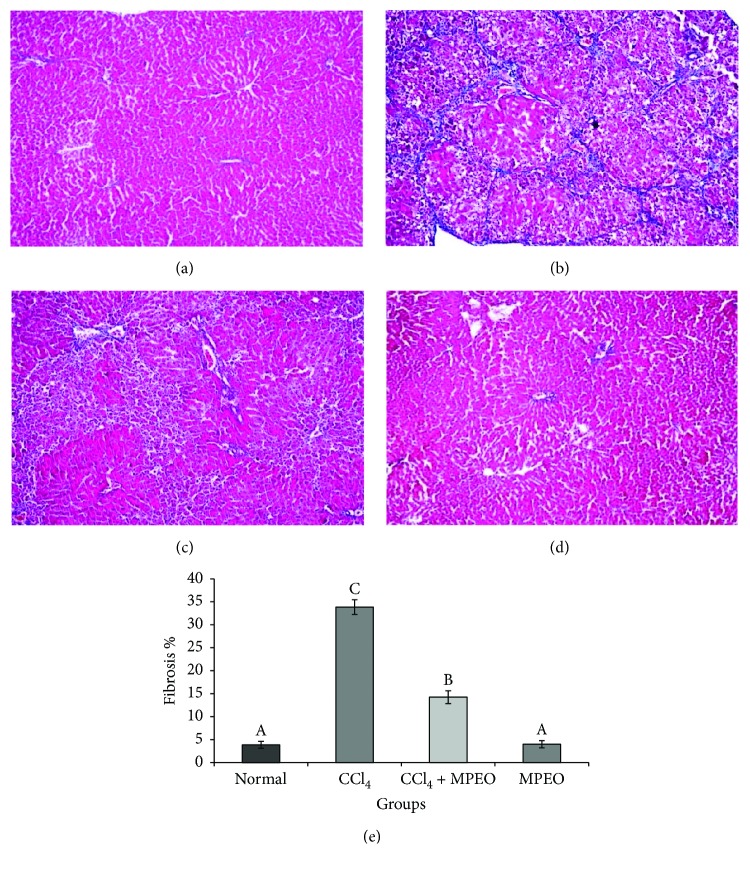
Photomicrograph of liver stained with MT stain (×100). (a and d) The normal control (group 1) and MPEO control (group 4) showing normal distribution of collagen fibers in the portal areas. (b) Group 2 (fibrosis control) showing marked fibrous bridging with excessive collagen fibers deposition. (c) Group 3 (MPEO-treated) showing marked attenuation of collagen fibers distribution and deposition. (e) Bar chart represents the hepatic fibrosis expressed as fibrosis %. Mean values with different superscripts are significantly different (*p* < 0.05).

The histopathological examination of the normal and MPOE groups revealed normal histological hepatic architecture (Figures 2(a) and 2(d)). The fibrosis control group (group 2) showed marked fatty degeneration of the hepatocytes, hepatocellular necrosis with mononuclear inflammatory cell aggregation, and collagen fibers bridging (Figure 2(b)). Group 3, receiving CCl_4_ + MPOE, showed marked attenuation of the previously described histopathological lesions compared to the fibrotic control group (Figure 2(c)). With MT staining, the liver of the control and MPEO groups showed normal distribution of collagen fibers (Figures 3(a) and 3(d)). The fibrotic control group showed severe bridging fibrosis with marked collagen deposition in the liver extending from portal to portal, portal to central, and central to central and formation of pseudolobules (Figure 3(b)). In group 3 (CCl_4_ + MPEO), the collagen deposition was markedly reduced, and the collagenous septa became thinner than those of the fibrotic control group (Figure 3(c)). The histopathological grading of liver fibrosis and the morphometric analysis of liver fibrosis % in different groups are shown in Table 5 and Figure 3(e), respectively, group 1 and 4 showed no significant difference in the liver fibrosis grading or fibrosis % (Figure 3(e) and Table 5). Group 3 (CCl_4_ + MPEO) showed significant reduction of the grading of liver fibrosis and fibrosis % compared to the fibrosis control group 2 as shown in Figure 3(e) and Table 5.

### 3.5. MPEO Regulates Profibrogenic Protein Expression in CCl_4_-Induced Fibrosis

**Figure 4:**
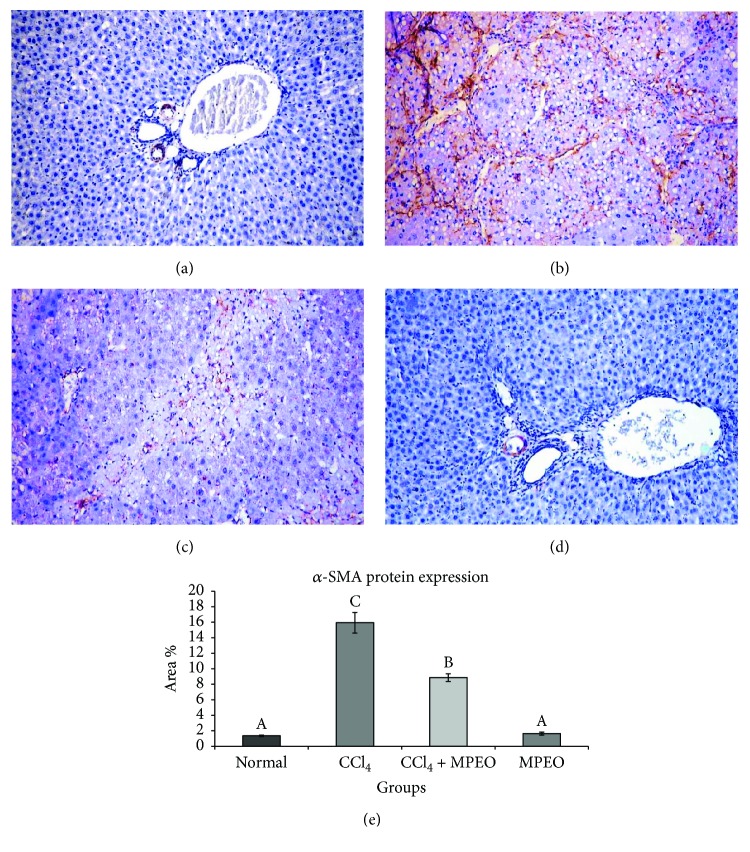
Representative *α*-SMA immunohistochemistry in liver tissues of the different experimental groups (×200). (a and d) The normal control (group 1) and MPEO control (group 4) showing *α*-SMA staining in the smooth muscle cells of the hepatic vessels. (b) Liver fibrosis control (group 2) showing strong immunostaining reaction in myofibroblast cells. (c) MPEO-treated (group 3) showing marked reduction in immunopositive reactive areas. (e) Bar chart represents the *α*-SMA immunohistochemistry expressed as area %. Mean values with different superscripts are significantly different (*p* < 0.05).

**Figure 5:**
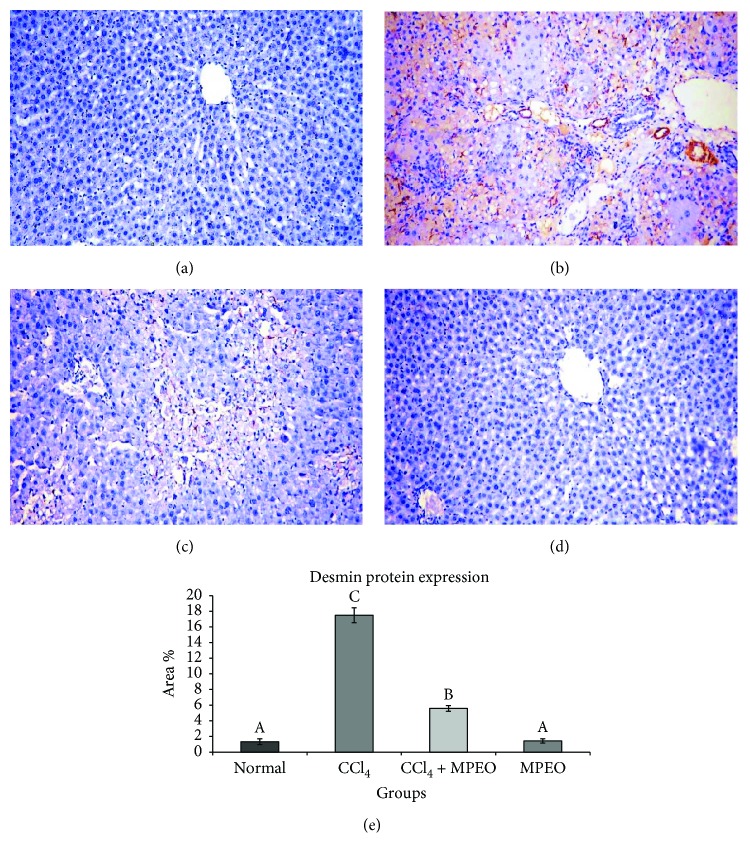
Representative desmin immunohistochemistry in liver tissues of the different experimental groups (×200). (a and d) The normal control (group 1) and MPEO control (group 4) showing very weak immunopositive reaction. (b) Liver fibrosis control (group 2) showing strong immunostaining reaction in perisinusoidal cells. (c) MPEO-treated (group 3) showing weak immunostaining of perisinusoidal cells. (e) Bar chart represents desmin immunohistochemistry expressed as area %. Mean values with different superscripts are significantly different (*p* < 0.05).

**Figure 6:**
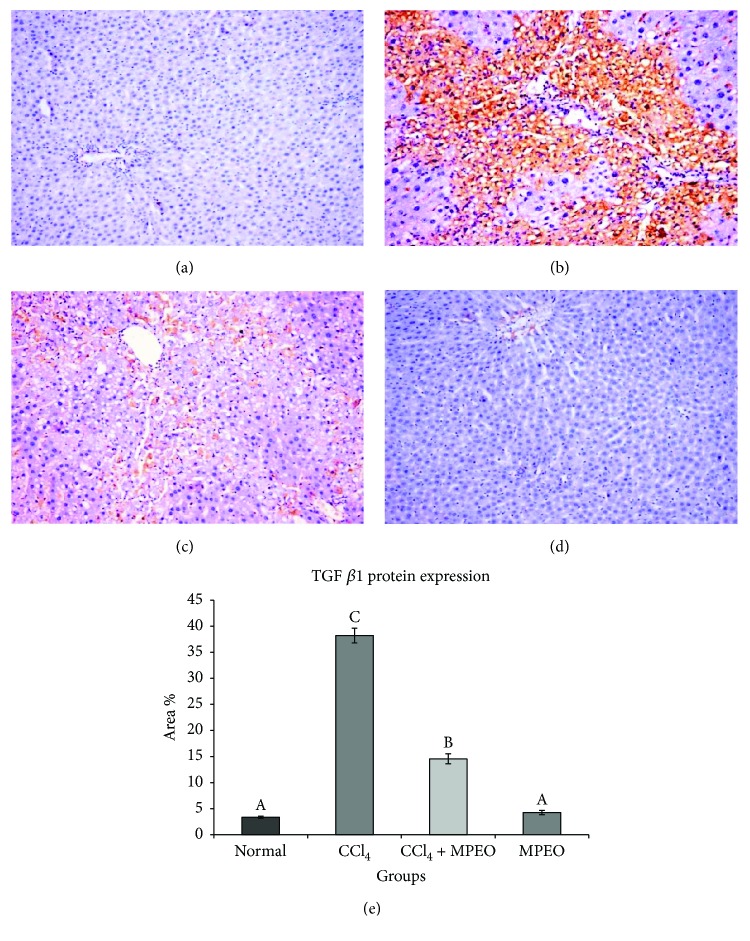
Representive TGF-*β*1 immunohistochemistry in liver tissue of the different experimental groups (×200). (a and d) The normal control (group 1) and MPEO control (group 4) showing weak immunopositive reaction in portal areas. (b) Liver fibrosis control (group 2) showing intense immunostaining in periductal cells in the portal tract, in perisinusoidal cells, around the blood vessels, and in sinusoidal lining cells. (c) MPEO-treated (group 3) showing mild reaction in some perisinusoidal cells. (e) Bar chart represents the TGF-*β*1 immunohistochemistry in liver expressed as area %. Mean values with different superscripts are significantly different (*p* < 0.05).

**Figure 7:**
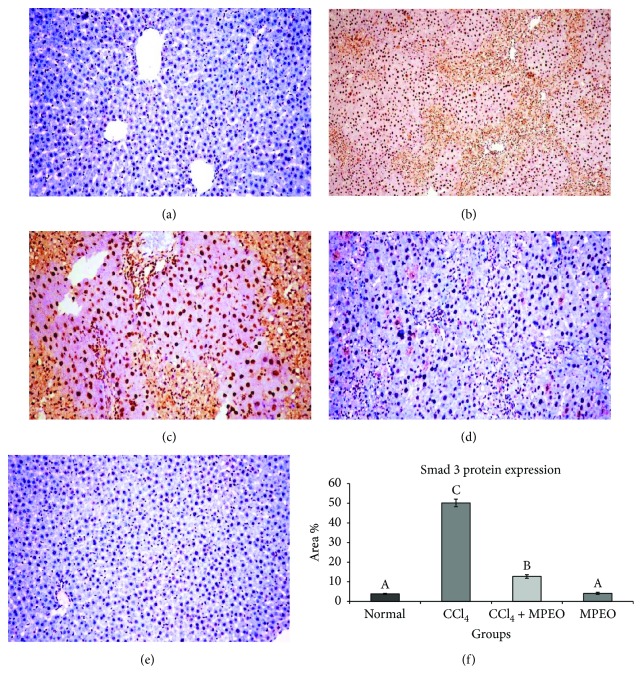
Representive SMAD3 immunohistochemistry in liver tissues of the different experimental groups. (a and e) The normal control and MPEO control (×200). (b) Liver fibrosis control (group 2) showing intense immunostaining reaction (×100). (c) Liver fibrosis control (group 2) showing strong cytoplasmic and nuclear staining (×200). (d) MPEO-treated (group 3) showing marked reduction of immunostaining reaction (×200). (f) Bar chart represents SMAD3 immunohistochemistry expressed as area %. Mean values with different superscripts are significantly different (*p* < 0.05).

*α*-SMA and desmin immunoreactivity appeared to be cytoplasmic and stained brown in colour. *α*-SMA expression was seen in smooth muscle cells of the blood vessels in the normal control and the MPOE control (Figures 4(a) and 4(d)). In the liver fibrosis control group, *α*-SMA staining located in the myofibroblast cells along collagenous septa bridging portal areas and central areas and desmin immunostaining was observed in perisinusoidal cells and interstitial myofibroblasts. *α*-SMA and desmin protein expression were significantly elevated in liver fibrosis control group than in the normal control (Figures 4 and 5). Group 3 (MPOE-treated) showed a significant reduction of *α*-SMA and desmin protein expression compared to the liver fibrosis control group (Figures 4 and 5). TGF-*β*1 was a cytoplasmic immunostaining, and it was observed in periductal cells in the portal tract of the normal control and MPOE control groups (Figures 6(a) and 6(d)). SMAD3 expression was nuclear and cytoplasmic immunostaining. The fibrotic control group showed TGF-*β*1 immunoreactivity in the periductal cells in the portal tract, in perisinusoidal cells, around the blood vessels, in sinusoidal lining cells, in inflammatory cells, and in the network around the necrotic hepatocytes; and a small amount was observed in necrotic hepatocytes. TGF-*β*1 and SMAD3 protein expression were significantly increased in the liver fibrosis control group than in the normal control (Figures 6 and 7). Group 3 (MPOE-treated) showed a sustained reduction of TGF-*β*1 and SMAD3 protein expression compared to the liver fibrosis control group (Figures 6 and 7, resp.). No significant difference was recorded between the normal control and MPOE control groups.

### 3.6. MPEO Downregulates p53 Expression in CCl_4_-Induced Fibrosis

**Figure 8:**
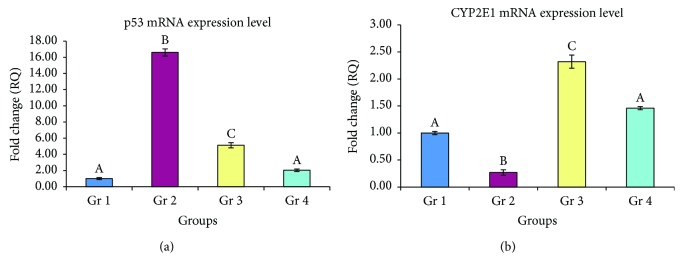
Quantitative real-time PCR of Tp53 and CYP2E1 mRNA in liver tissues of the different experimental groups. Values are expressed as means ± SD. GAPDH was used as an invariant housekeeping control gene for calculating the fold changes (RQ) in mRNA levels. Mean values having different letters are significantly different (*p* < 0.05).

**Figure 9:**
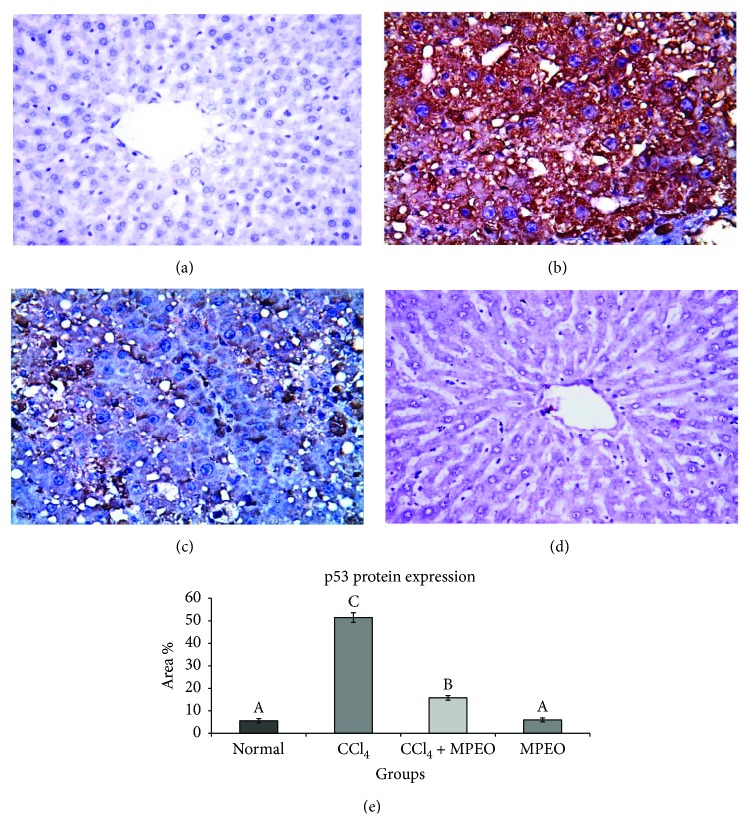
Representive p53 immunohistochemistry in liver tissues of the different experimental groups (×400). (a and d) The normal control and MPEO control very weak immunopositive reaction. (b) Liver fibrosis control (group 2) showing intense immunostaining in most of the hepatic cells. (c) MPEO-treated (group 3) showing immunopositive reaction in some hepatic cells. (e) Bar chart represents the p53 immunohistochemistry in liver tissues expressed as area %. Mean values with different superscripts are significantly different (*p* < 0.05).

Tp53 gene expression level showed a significant elevation in the liver fibrosis control (group 2). Tp53 mRNA level reached to about 16-folds of the control level. This CCl_4_-induced overexpression of Tp53 was markedly suppressed in MPOE-treated rats to about 2-fold as compared to that in the control (Figure 8(a)). At the protein level, p53 immunoreactivity was significantly increased in the liver fibrosis control. Group 3 treated with CCl_4_ + MPOE showed significantly decreased p53 immunoreactivity (Figure 9(c)).  Figure 9(e) summarizes the IHC analysis of p53 protein expression in the different groups. Nonsignificant differences in p53 mRNA and protein levels were detected between groups 1 and 4 (Figures 8(a) and 9), respectively.

### 3.7. MPEO Restored CYP2E1 Expression in CCl_4_-Induced Liver Fibrosis

Quantitative RT-PCR analysis showed a significant decrease in CYP2E1 mRNA in the liver fibrosis control group to 27% of that of the control group. MPOE-treated rats in group 3 showed marked increase in mRNA level to +2.32-folds of the normal level (Figure 8(b)).

## 4. Discussion

Fibrogenesis is a multicellular wound healing process that occurs as a frequent consequence of many chronic liver injuries [1]. Regardless of its etiology, hepatic fibrosis is generally characterized by oxidative tissue damage, inflammatory cells infiltration, HSCs activation, and excessive collagen deposition [31, 32]. Accumulating clinical and experimental evidences have shown that the halting of the fibrogenic process may allow the reversal of liver fibrosis. Indeed, resolution of liver fibrosis or even cirrhosis may occur upon eradication of the causative insult [33, 34]. Oxidative stress has been proposed as a conjoint pathological mechanism in the initiation and progression of fibrosis [3]. Thereby, inhibition of ROS-mediated fibrogenesis might yield great potential therapeutic benefits. In this context, attention is progressively shifting towards herbal products and their antioxidant constituents [3, 11]. *M. piperita* has been known for its great multipharmaceutical benefits [35, 36]. The active phytocompounds of MPEO contribute to many of its profound therapeutic actions. Although the hepatoprotective effect of MPOE was previously demonstrated [18, 19], its antifibrogenic influence against hepatic fibrosis has not been investigated yet. The present study aimed to evaluate the potential of MPOE to ameliorate CCl_4_-induced hepatic fibrosis in rats and to elucidate some aspects of its underlying molecular mechanisms.

MPEO was reported to have a wide range of components, including menthol, menthone, menthofuran, and methyl acetate, and other pharmacologically active compounds such as flavonoids, tannins, and caffeic acid [37]. In the current study, the chemical profile of MPEO, as presented in Table 2, is characterized by the dominant presence of the oxygenated monoterpenes menthol (46.7%) and menthone (18.3%). A high percentage of carvone (15%) was observed compared to that of the composition previously reported by Sun et al. [38]. Different chemotypes of *M. piperita* were reported in other countries in which the major constituent in MPEO is linalool as in *M. piperita* collected from Brazil [39] or limonene as in *M. piperita* collected from India [40]. This diversity in the chemical composition may be attributed to geographical and soil condition, biosynthetic factors, and collection time [38]. However, the composition of MPEO presented in the current study still maintains a certain level of chemosimilarity with some previously reported MPEO compositions [38]. Mentha species are known for their ability to exhibit strong antioxidant and radical scavenging activities owing to the presence of valuable secondary metabolites in the essential oil and phenolic substances [41]. Previous studies showed that the radical scavenging activity of essential oil of *M. piperita* species is attributed to the presence of menthone and menthol, with the presence of the hydroxyl radical (^•^OH). The vast majority of these antioxidants (Figure 1) have the ability to trap the free radicals and interrupt the chain reaction due to the presence of at least one aromatic ring in their structures [42].

CCl_4_-induced fibrosis has been extensively used as an *in vivo* model for the study of liver damage [14, 43, 44]. CCl_4_ toxicity represents a multifactorial process involving its detoxification by CYP450 into the highly reactive CCl_4_-derived free radicals, covalent binding to macromolecules, stimulation of inflammatory cytokines, oxidative damage with subsequent necrosis of hepatocytes, and activation of HSCs [44].

Fibrogenesis is mediated by a complex interplay of signaling pathways. Of particular note, TGF-*β*1/SMAD is one of the core mechanisms of fibrogenesis [45]. Recently, much information has emerged concerning the central role of TGF-*β*1 as the principal driver of excessive scarring and tissue fibrosis. TGF-*β*1, in HSCs, acts by stimulating collagen I expression and inhibiting ECM degradation [4, 46]. Excessive TGF-*β*1 release by necrotic hepatocytes is considered as one of the first signals to adjacent quiescent HSCs to be activated by transdifferentiation into myofibroblasts-like cells [47]. Upon activation, TGF-*β*1 binds to its cell-surface receptor complexes and initiates an intracellular signaling cascade resulting in phosphorylation of SMAD2 and SMAD3. Subsequently, the activated SMAD2 and SMAD3 form stable oligomer complexes with SMAD4. These complexes actively shuttle into the nucleus to regulate the transcription of target genes [48, 49]. It has been demonstrated that SMAD3 is a key element in TGF-*β*1–induced fibrosis [50]. A number of fibrogenic genes (e.g., collagens) and markers (e.g., *α*-SMA) are SMAD3-dependent as SMAD3 directly binds to the DNA regulatory sequences of these target genes [51, 52]. Moreover, SMAD3 inhibits matrix metalloprotease 1 activity in fibroblasts and activates tissue inhibitor of metalloproteases and thus inhibits ECM degradation [53]. Considering this major role of TGF-*β*1 signaling in the pathobiology of liver fibrosis, TGF-*β*1 or its downstream mediators may provide important targets for the new therapeutic strategies of liver fibrosis [5].

In the present study, eight weeks of CCl_4_ administration was sufficient to induce severe hepatotoxic changes detected by the considerably elevated activities of serum ALT and AST markers (Table 3). Increased release of these cytosolic enzymes in the serum reflects hepatocyte damage and leakage and provides important diagnostic biomarkers for hepatic diseases [54]. Moreover, high AST and ALT levels are associated with an increased risk of fibrosis progression [55]. MPEO administration reversed the elevated levels of ALT and AST compared to those of the CCl_4_ group. These findings are consistent with those of previous studies [18, 19].

LPO is thought to be the initiation step of CCl_4_ hepatotoxicity [56]. Lipid hydroperoxides are unstable and so degrade rapidly into a variety of secondary metabolites such as MDA, 4-hydroxynonenal (4-HNE), and other conjugated dienes [57]. MDA serves as a main biomarker to assess the level of lipoperoxidative tissue damage [58]. In accordance with previous studies [59, 60], a significant increase in liver MDA content was observed in the CCl_4_ group, suggesting an enhanced LPO (Table 3).

Furthermore, CCl_4_-intoxicated rats showed a significant increase in the liver NO (Table 3). This elevated NO indicates the stimulation of inflammatory cells and release of inflammatory cytokines [61]. NO participates in the induction of fibrosis through its reaction with superoxide anions forming highly reactive peroxynitrite radicals, which induce HSCs activation and accelerate the progression of liver fibrosis [56]. The obtained results closely agreed with those mentioned by Sagor et al. [62] and Abdel Salam et al. [63], who suggested the correlation between the hepatic NO content and the degree of liver fibrosis.

On the other hand, a significant decline in the liver antioxidant capacity was detected in the CCl_4_ group evidenced by the decreased SOD and CAT activities and the depletion in GSH content (Table 4). This disturbance in the prooxidants/antioxidants balance was further verified by the low TAC (Table 4).

Histopathological analysis of liver sections provided an initial evidence of CCl_4_-induced liver hepatocellular damage and fibrosis (Figure 2 and Table 5). Degenerative changes including fatty degeneration, congestion, and cytoplasmic vacuolization were observed in the liver fibrosis control group (Figure 2(b)). These findings were in agreement with those obtained by Ogaly et al. [14] and Yacout et al. [64]. Additionally, MT staining revealed clear fibrotic bridging in the liver sections of group 2 (Figure 3(b)).

Immunohistochemical analysis of desmin, a hallmark of both quiescent and activated HSCs, revealed a significant elevation in desmin-immunopositive cells in the livers of CCl_4_-injured group 2 (Figure 5(b)). In accordance with Fujii et al. [65], this finding implicates increased numbers of HSCs in the livers of CCl_4_-treated animals, as expression of desmin together with the stellate-shaped morphology is the main characteristic feature of HSCs [66].

Another important evidence for the activation of HSCs and progression of fibrosis was the significantly increased number of *α*-SMA-immunopositive cells in the CCl_4_ fibrosis group (Figure 4(b)), as compared to the control group (Figure 4(a)). *α*-SMA is a reliable hallmark for HSCs activation into the myofibroblastic phenotype and considered as an important myogenic marker that plays a significant role in collagen I deposition by activated HSCs [67]. These obtained findings, in accordance with Rockey et al. [68], indicate that CCl_4_ stimulated HSCs activation and transdifferentiation. Moreover, it was found that SMAD3 increases *α*-SMA production and stimulates its organization into stress fibers [69].

In the current study, the livers of group 2 showed an augmented expression of TGF-*β*1 and SMAD3 proteins as detected by the increased immunoreactivity compared to those of the control group (Figures 6 and 7). Similar findings were previously reported in rats [44].

MPOE at the investigated dose (50 mg/kg) significantly improved the liver indices, antioxidant profile, and histological picture of liver tissue. MPOE significantly reduced serum ALT and AST, indicating a preserved liver integrity and improved function (Table 3). MPOE treatment markedly increased the liver antioxidant capacity by restraining the LPO byproduct (MDA) and simultaneously enhancing the activities of SOD, catalase, and regeneration of GSH (Table 4). Our data come in line with previous studies that reported the hepatoprotective effect of *M. piperita* [18] or its oil [19]. It was suggested that several active components of Mentha have antioxidant and antiperoxidant activities that provoke the capacity of endogenous antioxidant systems and protect against toxic hepatic damages [18, 19, 70].

Coadministration of MPOE with CCl_4_ significantly improved the liver histopathology (Figure 2) and reduced the fibrotic changes (Figure 3, Table 5). Furthermore, the MPOE-treated group showed a marked reduction in both *α*-SMA- and desmin-immunopositive cells (Figures 4(c) and 5(c)). These data reflect the efficacy of MPOE to reduce the hepatocellular toxic effects of CCl_4_ and to suppress HSCs activation and proliferation as well.

In the same context, MPOE-treated group 3 showed marked reductions in TGF-*β*1 and SMAD3 proteins expression (Figures 6(c) and 7(c)) compared to the CCl_4_ fibrosis group (Figures 6(b) and 7(b)). According to Kanzler et al. [47] and Chen et al. [71], the observed suppression in TGF-*β*1 protein expression and the subsequent downregulation of SMAD3 in the livers of group 3 could explain the observed reduction of *α*-SMA-positive cells (Figure 4(c)), collagen deposition (Figure 3(c)), and fibrosis % (Figure 3(e)) and the reduced fibrosis scores (Table 5) in this group in comparison with the liver fibrosis control group. These findings support the antifibrogenic potential of MPOE against CCl_4_-induced liver fibrosis.

p53 is a tumor-suppressor protein that regulates the transcription of a plethora of target proteins involved in the cell cycle, differentiation, and apoptosis [72]. In normal redox state, p53 expression and degradation are tightly regulated by a variety of proteins to maintain a low level of p53 [73]. Different stress signals including ROS, hypoxia, and oncogene activation induce p53 that becomes transcriptionally active, leading to cell cycle arrest, DNA repair, and apoptosis [8, 9]. It was reported that hepatocyte p53 activation resulted in spontaneous liver fibrosis and induced hepatocyte apoptosis, in addition to upregulation of connective tissue growth factor (CTGF) [74]. *In vitro* study showed that p53 induces CTGF in hepatocytes that occur via regulation of microRNA [75].

Recent studies highlighted the possible involvement of p53 in fibrogenesis as a profibrotic mediator [8, 9, 74]. p53 has been implicated as an inducer of several profibrotic effectors such as TGF-*β*1, CTGF, *α*-SMA, and fibronectin [76]. During the fibrogenic process, a crosstalk between p53 and TGF-*β*1 has been suggested to stimulate transcription of the fibrogenic target genes [77, 78]. Under oxidative stress, excessive ROS induces various transcription factors such as p53, activator protein-1 (AP-1), and NF-*κ*B by altering the DNA binding sites or by oxidizing the cysteine residues of such proteins resulting in conformational change of their tertiary structure with subsequent protein degradation activation or inhibition. Activated p53 plays a crucial role in inducing apoptosis to prevent the propagation of DNA damage [72].

Few reports on the role of p53 and TGF-*β*1 and their interplay in the mechanism of fibrogenesis have arisen [75–78]. This encouraged our team to study the possible role of p53 and TGF-*β*1 in MPEO antifibrogenic mechanism. In the current investigation, Tp53 mRNA level showed a significant increase in response to CCl_4_ fibrosis (Figure 8(a)). Consequently, a considerable level of p53 protein was detected in the livers of the fibrosis group (Figure 9). These findings agreed with those of previous studies that reported that p53 expression increases following the onset of liver injury [8, 9]. In the MPOE-treated group, both Tp53 mRNA (Figure 8(a)) and p53 protein expression (Figure 9(c)) were significantly suppressed. This p53 downregulation might have contributed to the reduced hepatic fibrosis in this group.

Pharmacological p53 inhibitors have been proposed as a therapeutic application to alleviate tissue fibrosis [8].

Analysis of CYP2E1 gene expression aimed to evaluate for the detoxifying capacity of the liver. CCl_4_ intoxication resulted in a significant suppression in CYP2E1 mRNA (Figure 8(b)). This specific CYP2E1 downregulation in CCl_4_ hepatic fibrosis was previously reported [18] and may have contributed to a direct attack of CYP2E1 transcript by the reactive CCl_4_ metabolites leading to its degradation. Besides, the inflammatory response associated with fibrosis could exert a suppressive effect on CYP2E1 expression [79]. Interestingly, MPOE treatment counteracted this CYP2E1 downregulation (Figure 8). This preservation of CYP2E1 expression could be one of the MPOE hepatoprotective and antifibrogenic actions [80]. These findings are consistent with those of previous studies [62] that reported the ability of peppermint to modulate both phase I and phase II liver drug-metabolizing enzymes. Besides, eugenol, an active component of MPOE (Table 2), has been previously shown to induce detoxification enzymes [18].

## 5. Conclusion

MPEO significantly ameliorates the severity of CCl_4_-induced liver fibrosis through improving the oxidative status and restoring hepatic CYP2E1 expression. These MPEO actions could be mediated by inhibiting TGF-*β*1/SMAD signaling proteins and downregulation of p53 at both gene and protein levels. These data suggest that MPEO might be an effective antifibrogenic agent in the prevention of liver fibrosis progression. MPEO could help in developing a promising approach against oxidation-caused liver fibrosis.

## Figures and Tables

**Table 1:** Primers sequences.

Gene	Primer sequence		Ref.
GAPDH	Forward	5′-ACCACAGTCCATGCCATCAC-3′	[81]
	Reverse	5′-TCCACCACCCTGTTGCTGTA-3′	

TP53	Forward	5′-TCCCTAAGTATCCTCAGTGA-3′	[82]
	Reverse	5′-GTAATCGAAGCGTTTGTTGA-3′	

CYP2E1	Forward	5′-TCCAGGTTTGCACCAGACTCT-3′	[14]
	Reverse	5′-TCCACCACCCTGTTGCTGTA-3′	

**Table 2:** Chemical composition of MPEO by GC/MS.

Retention time	Compound	Relative %
4.8	Limonene	0.78
5.1	1,8-Cineol	1.35
6.8	*p*-Menthone	18.30
7.6	Beta pinene	1.10
8.2	*iso*-Menthone	5.31
8.4	Menthol	46.70
8.9	Pulegone	6.30
8.92	d-Camphor	3.30
9.1	l-Carvone	15.20
12.8	*trans*-Caryophyllene	0.95
	Total	99.35

**Table 3:** Effects of MPEO on liver enzymes, NO content, and lipid peroxidation byproduct (MDA) in CCl_4_-induced liver fibrosis.

Experimental groups	ALT (U/L)	AST (U/L)	MDA (*μ*M/g tissue)	NO (mmol/L)
Group 1	18.4 ± 0.84	46 ± 1.52	60.8 ± 2.47	3.98 ± 0.32
Group 2	1147.2 ± 42.25	1088 ± 65.73	143.8 ± 3.97	16.21 ± 0.89
Group 3	882 ± 83.19	657 ± 31.74	89.14 ± 3.16	7.11 ± 0.72
Group 4	34 ± 1.12	69 ± 1.47	71.32 ± 2.36	5.42 ± 0.68

Group 1: normal control; group 2: liver fibrosis control; group 3: MPEO-treated; group 4: MPEO control. Values are expressed as mean ± SE. Different superscripts mean significant differences between groups in the same column at *p* < 0.05. ALT: alanine transaminase; AST: aspartate transaminase; NO: nitric oxide; MDA: malondialdehyde.

**Table 4:** Effects of MPEO on the hepatic antioxidant profile in CCl_4_-induced liver fibrosis.

Experimental groups	GSH (*μ*M/g liver)	SOD (U/mg protein)	CAT (U/mg protein)	TAC (*μ*mol/g liver)
Group 1	12.48 ± 0.32^a^	82.95 ± 2.56^a^	2.15 ± 0.07^a^	5.24 ± 0.25^a^
Group 2	2.93 ± 0.14^b^	20.29 ± 1.13^b^	0.93 ± 0.06^b^	2.08 ± 0.16^b^
Group 3	4.8 ± 0.19^c^	47.6 ± 1.22^c^	1.37 ± 0.05^c^	3.88 ± 0.12^c^
Group 4	11.6 ± 0.31^a^	72.45 ± 2.10^a^	1.94 ± 0.04^a^	4.30 ± 0.22^a^

Group 1: normal control; group 2: liver fibrosis control; group 3: MPEO-treated; group 4: MPEO control. Values are expressed as mean ± SE. Different superscripts mean significant differences between groups in the same column at *p* < 0.05. GSH: reduced glutathione; SOD: superoxide dismutase; CAT: catalase; TAC: total antioxidant capacity.

**Table 5:** Effect of MPEO on the pathological grading of CCl_4_-induced fibrotic liver in rats.

Group	*n*	Pathological grading of hepatic fibrosis							*p* value
		0	I	II	III	IV	V	VI	
Group 1	7	7	0	0	0	0	0	0	—
Group 2	7	0	0	0	2	4	1	0	0.00^a^
Group 3	7	0	3	3	1	0	0	0	0.011^b^
Group 4	7	7	0	0	0	0	0	0	—

Group 1: normal control; group 2: liver fibrosis control; group 3: MPEO-treated; group 4: MPEO control. Data are presented as the mean of ten fields. *n*: number of rats. ^a^Significant difference from the control group at *p* < 0.01. ^b^Significant difference from model group at *p* < 0.05.
